# The use of BokaFlo™ instrument to measure salivary flow

**DOI:** 10.1186/s12903-021-01477-4

**Published:** 2021-04-12

**Authors:** Braden S. Fallon, Trevor J. Chase, Elaine M. Cooke, Amir Ghazitabatabaei, Nathan O. Naylor, Jordan J. Cutshall, Bryan G. Trump, Melodie L. Weller

**Affiliations:** School of Dentistry, University of Utah, Salt Lake City, UT USA

**Keywords:** BokaFlo, BokaFlo™, Passive Drool Test, Xerostomia, Hyposalivation, Saliva Flow, Dry mouth, Sjogren’s syndrome, Testing saliva flow, Sialometry

## Abstract

**Background:**

Dry mouth currently affects roughly 20% of the population and is a condition characterized by chronic hyposalivation and/or subjective reports of xerostomia. Low saliva flow can be indicative of other undiagnosed diseases, such as primary Sjogren’s syndrome, and may contribute to difficulty chewing, increased caries susceptibility and infection. The passive drool test (PDT) is the primary method used to evaluate patients for hyposalivation but it is time-consuming and inconvenient. New methodology is needed to facilitate increased testing for hyposalivation in the dental clinic. The aim of this study was to evaluate an alternative method to measure salivary flow in dental offices.

**Methods:**

In this study, we tested a new biomedical device, the BokaFlo™, to measure salivary flow in subjects in comparison to the current PDT standard. Participants completed an oral health questionnaire and saliva flow was evaluated by the PDT and the BokaFlo™ system.

**Results:**

Saliva flow as measured by the BokaFlo™ positively correlated with the saliva flow measured by the PDT methodology (r = 0.22, *p* < 0.05). The device predicted low saliva flow in subjects with a sensitivity of 0.76 and specificity of 0.84 for subjects with hyposalivation, defined as a saliva flow rate of ≤ 0.1 ml/min. A significant negative correlation between the total oral health questionnaire score and the likelihood of participant exhibiting low salivary flow was observed (r = − 0.31, *p* < 0.006).

**Conclusion:**

The BokaFlo™ was effectively able to measure low saliva flow correlating with the PDT methodology and may provide more efficient testing of saliva flow in the dental office.

**Supplementary Information:**

The online version contains supplementary material available at 10.1186/s12903-021-01477-4.

## Background

Published studies estimate that 20% of the population suffers from dry mouth with the prevalence increasing with age [1, 2]. Saliva is integral to many important functions of the oral cavity and chronic changes to saliva flow can significantly impact the patients’ oral health. The passive drool test (PDT) is the current gold standard for assessment of decreased saliva flow, but this test is often not performed in the dental clinic due to the time-intensive nature of the procedure [3]. Alternative methods for measuring saliva flow in the dental office have not been thoroughly explored to be adopted into standard practice. Therefore, a study was designed to evaluate the ability of a new device, the BokaFlo™ instrument, to efficiently and simply measure saliva flow in the dental office.

Changes to saliva flow and/or saliva composition can significantly impact the patients’ long-term oral health. As a fluid, saliva buffers the pH changes induced by bacterial metabolism which helps to prevent dental caries formation [4–8]. Patients with hyposalivation frequently experience painful symptoms due to the abrasion of the teeth or food against the soft mucosa and tongue. Furthermore, hyposalivation impairs bolus formation during mastication, causes difficulty swallowing, and favors induction of dysgeusia resulting from damage to the taste buds [9, 10]. Reduction in saliva flow may significantly impact the function of innate immunity within the oral cavity resulting in increased oral and systemic infections [7, 9, 11, 12]. Therefore, monitoring patients for hyposalivation is essential for early detection, diagnosis, and treatment of oral and systemic diseases [8, 13].

The PDT is currently the primary method used to detect deficits in saliva flow. Although alternative methods of measuring stimulated or unstimulated saliva flow have been explored [14–16], no other single methodology to measure saliva flow has been readily adopted. For the PDT, subjects are asked to tilt their head forward to allow saliva to pool and passively drool into a collection vial for 5–20 min. While the instructions for PDT are simple to follow and non-invasive [3, 16], the use of PDT in the dental office is limited due to the length of time for the procedure, training of clinic staff, and patients’ discomfort with the procedure. There is a significant need for simple and efficient methods for saliva collection in the dental clinic.

The aim of this study was to evaluate an alternative method to measure salivary flow and make saliva flow assessment in the dental clinic more accessible. To accomplish this, participants completed a scored oral health questionnaire, PDT, and assessment of saliva flow by a new salivary flow measurement device, the BokaFlo™. Measured saliva flow results from the BokaFlo™ were analyzed and compared to an oral health questionnaire and PDT results. Our study demonstrates a significant correlation between the BokaFlo™ instrument and the PDT in measuring hyposalivation and represents a new, time-efficient, and accessible method for testing saliva flow in the dental clinic.

## Methods

### Subject population

This prospective, cohort study was carried out in the dental clinic at the University of Utah, School of Dentistry. The study was approved and completed in accordance with the Institutional Review Board (IRB) program coordinated by the University of Utah's Office of Research Integrity and Compliance (ORIC) (IRB 00118693, “Evaluation of Saliva Flow Using BokaFlo™ Device in Drool Test”).

**Fig. 1:**
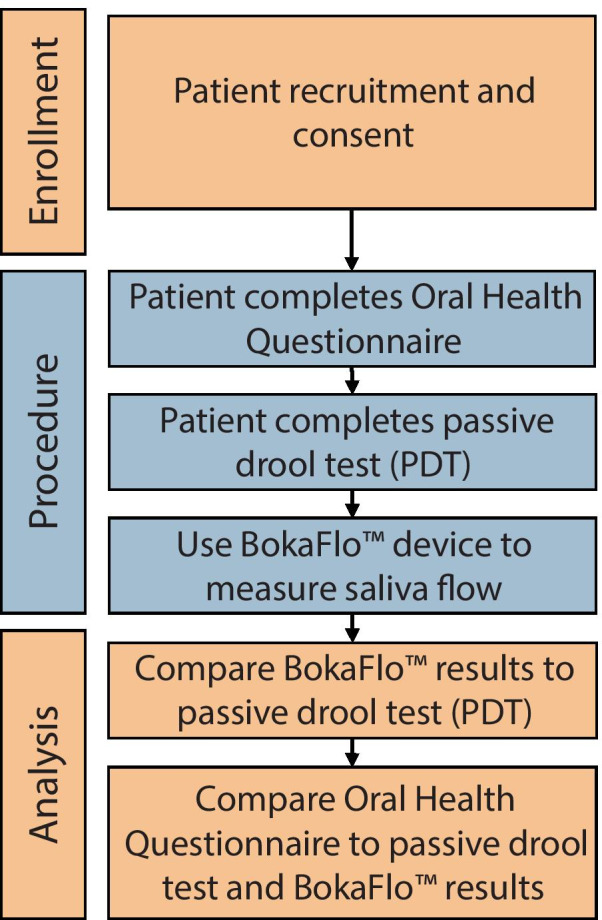
Outline of study illustrating the enrollment process, performed procedure, and data analysis

In total, 79 participants were enrolled in the study from patients seeking dental treatment at the School of Dentistry and volunteers from the public. Written consent was obtained for all participants meeting inclusion criteria for study. Each participant was asked to complete the Oral Health Questionnaire, the passive drool test and measure saliva flow using the BokaFlo™ instrument as outlined in Fig. 1. This study followed the recommendations of the Strobe guidelines [17].

Inclusion criteria:

1. Age 18–99 years
2. Willing to complete short survey on xerostomia symptomology
3. Willing to complete a 5 min passive drool test
4. Willing to complete the BokaFlo™ test

Exclusion criteria:

1. Chewing gum, taking a breath mint, brushing teeth or eating within 15 min prior to administering the test
2. Active mouth ulcers and lesions
3. Any cognitive or physical condition that prohibits completion of the test or the survey

### Questionnaire

Participants were administered the oral health questionnaire to inquire about age, sex, timing of last food and liquid consumption, previous diagnosis of hyposalivation, whether they had been prescribed any medications, and whether they believed they had healthy saliva flow (Additional file 1: Fig. S1). The oral health questionnaire also requested information from each participant to assess subjective dry mouth/xerostomia symptomology in addition to oral and general health questions including whether they had experienced bleeding gums, cavities, bad breath, frequent cough, frequent colds, and tooth sensitivity in the last 12 months.

### Passive drool test (PDT)

**Fig. 2:**
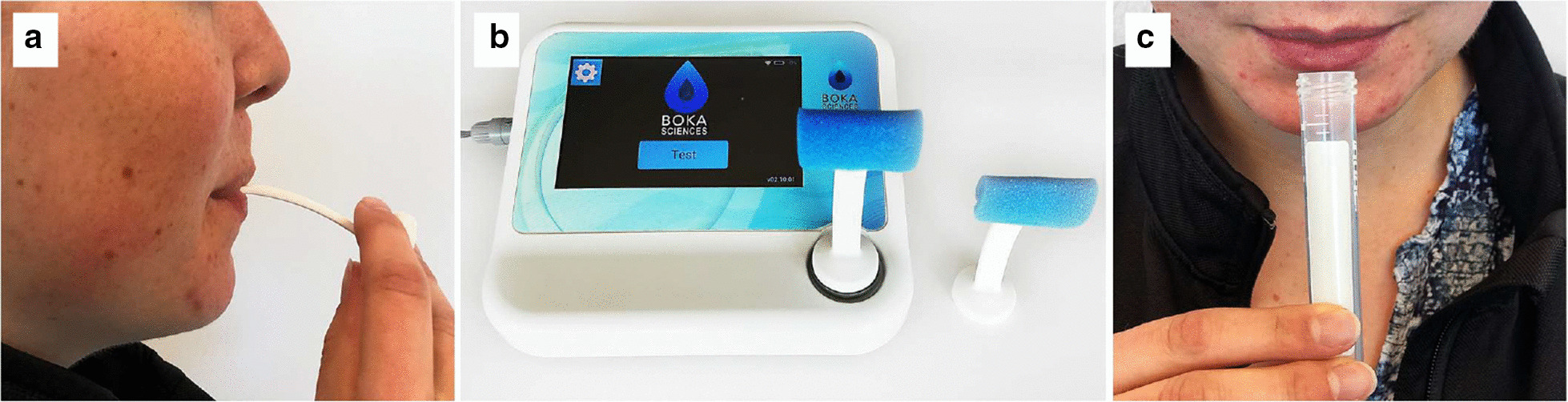
Saliva collection protocol for the BokaFlo™ and passive drool test (PDT). Saliva collection protocol demonstrating methodology used to assess subject salivary flow as measured with BokaFlo™ and PDT. **a** Participants cleared their mouth of any existing saliva, opened their mouth and the BokaFlo™ disposable device was placed under the participant's tongue for 3 s. **b** The disposable device was placed on the BokaFlo™ instrument, removed, and another disposable device placed on the instrument to tare. Subjects were then asked to allow saliva to pool in mouth for 60 s. The second BokaFlo™ disposable device was placed under subjects’ tongue for 3 s and then placed on BokaFlo™ instrument. BokaFlo™ instrument displays salivary flow as milliliters/minute (ml/min). **c** The PDT was performed by first having subject clear mouth of saliva, then allowing saliva to passively flow into the saliva collection tube for 5 min while sitting upright and with their head tilted forward and down. Resulting PDT rendered saliva flow as ml/min

The passive drool test (PDT) was performed as previously detailed (Fig. 2) [3, 14]. Participants in the study were asked to clear the mouth of any existing saliva by gathering saliva and swallowing. Participants were then asked to allow saliva to passively flow into the saliva collection tube for 5 min while sitting upright and with their head tilted forward and down. Participants were asked to refrain from speaking or swallowing during the 5-min saliva collection period. Sample volumes were measured and the volume of saliva flow per minute was calculated (ml/min).

### BokaFlo™ testing

The BokaFlo™ protocol was performed as outlined in Fig. 2. Participants who had completed the PDT were asked to clear the mouth of any existing saliva. A staff member then directed the participants to open their mouth and the first BokaFlo™ disposable device was placed under the participant's tongue. The staff then instructed the participant to close their mouth for 3 s to collect any remaining saliva. The device was then removed, placed onto the BokaFlo™ instrument for the initial reading, and then discarded. A second BokaFlo™ disposable device was then placed on the BokaFlo™ instrument to tare the device. Participants were instructed to allow saliva to pool under the tongue with the head tilted forward for 60 s while refraining from speaking or swallowing. After 60 s, the second BokaFlo™ disposable device was then placed under the participant's tongue for 3 s with the participants’ mouth closed. It was then removed and placed on the BokaFlo™ instrument for the final measurement. The BokaFlo™ instrument displayed the calculated saliva flow as the volume of saliva per minute (ml/min). One possible concern is that the BokaFlo™ may stimulate saliva flow. However, prior experiments have demonstrated that the addition of an object in the oral cavity, such as a swab, does not significantly alter salivary flow [14]. Therefore, the PDT and BokaFlo™ tests can be compared as unstimulated salivary flow measurement methods. As with the PDT, low saliva flow is defined as ≤ 0.1 ml/min, > 0.1 ml/min to ≤ 0.3 ml/min is defined as borderline hyposalivation, and > 0.3 ml/min is defined as normal or healthy saliva flow [18, 19].

### Statistical data analysis

A Pearson correlation was performed between the matched PDT and BokaFlo™ saliva flow measures and between each questionnaire response and the BokaFlo™ and PDT saliva flow results. Population summary statistics and statistical analysis of difference of means (unpaired t-test) for total questionnaire score for PDT and BokaFlo™ were performed in Prism 8 (GraphPad Software, United States).

## Results

**Fig. 3:**
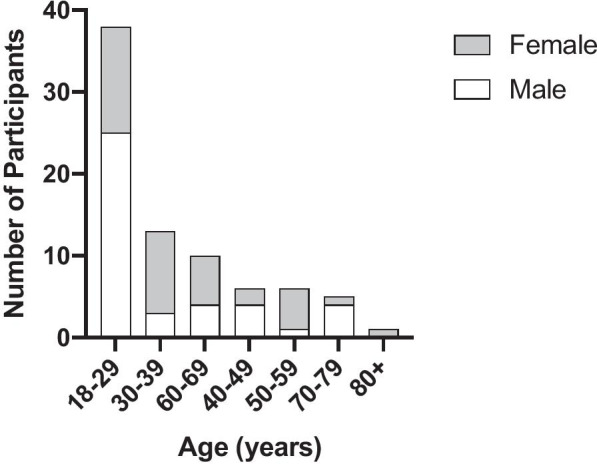
Demographics of study participants. The cohort of 79 participants consisting of 41 males and 38 females took part in this study. The age of participants ranged from 18 to 80 years of age with an average participant age of 39.20 ± 16.87 years of age

Participants were recruited and consented according to IRB guidelines and completed the study questionnaire of reported xerostomia. The salivary flow was measured with both the standard passive drool test (PDT) and the BokaFlo™. The saliva collection protocols demonstrating how subjects had their salivary flow measured with the PDT and BokaFlo™ are depicted in Fig. 2. A cohort of 79 participants, which consisted of 41 males and 38 females, was analyzed. The age of participants ranged from 18 to 80 years of age with the average participant age of 39.20 ± 16.87 years of age (Fig. 3).

### Saliva Flow Analysis by PDT and BokaFlo™

**Fig. 4:**
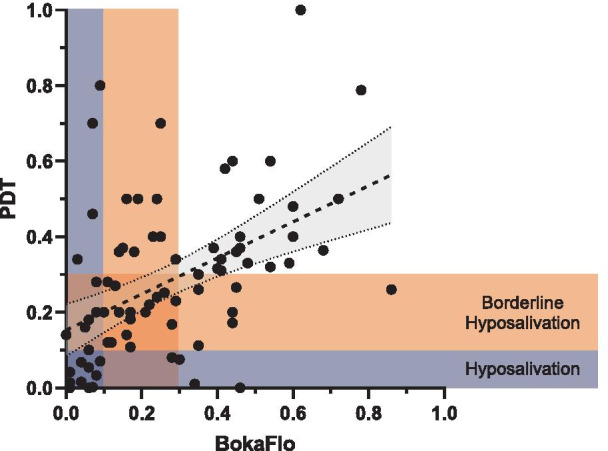
Unstimulated saliva flow measured by BokaFlo™ correlates with PDT. Passive drool test (PDT) identified 45 (56.9%) participants with saliva flows ≥ 0.3 ml/min, the lower limit of normal salivary function. 17 (21.5%) participants had measured PDT results of less than or equal to 0.1 ml/min, the clinical range for hyposalivation. BokaFlo™ identified 50 (63.3%) participants with saliva flows ≥ 0.3 ml/min, the lower limit of normal salivary function. 23 (29.1%) participants had measured PDT results of less than or equal to 0.1 ml/min, the clinical range for hyposalivation

Saliva flow categorization was characterized as previously published by Villa et al. [18]. The PDT identified 17 participants with hyposalivation (≤ 0.1 ml/min), 28 participants with borderline hyposalivation (> 0.1 ml/min to ≤ 0.3 ml/min), and 34 participants with normal saliva flow (Fig. 4, Table 1). The BokaFlo™ test identified 23 participants with hyposalivation (≤ 0.1 ml/min), 27 participants with borderline hyposalivation (> 0.1 ml/min to ≤ 0.3 ml/min), and 29 participants with normal saliva flow. The sensitivity and specificity of the BokaFlo™ methodology as compared to the PDT methodology was 0.84 and 0.76, respectively (Table 1). The goodness of fit for the linear regression between the two tests was r = 0.22 and *p* < 0.05.

**Table 1:** Hyposalivation detected in 29.1% of participants with the BokaFlo™ instrument

	PDT	BokaFlo™
Hyposalivation (≤ 0.1 mL/min)	17 (21.5%)	23 (29.1%)
Borderline Hyposalivation (0.1 < x ≤ 0.3)	28 (35.5%)	27 (34.2%)
Normal (> 0.3 mL/min)	34 (43.0%)	29 (36.7%)

### Oral Health Questionnaire vs BokaFlo™ flow

**Fig. 5:**
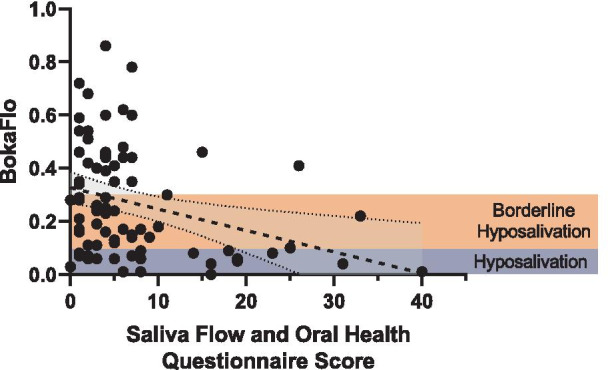
Oral Health Questionnaire identified a significant, negative correlation to BokaFlo™ measurement. Pearson correlation analysis comparing survey results to the BokaFlo™ indicated a significant negative correlation between the total questionnaire score, equating to increased oral health symptomology, and the likelihood of participant exhibiting low saliva flow (r = − 0.31, *p < 0.006)

**Fig. 6:**
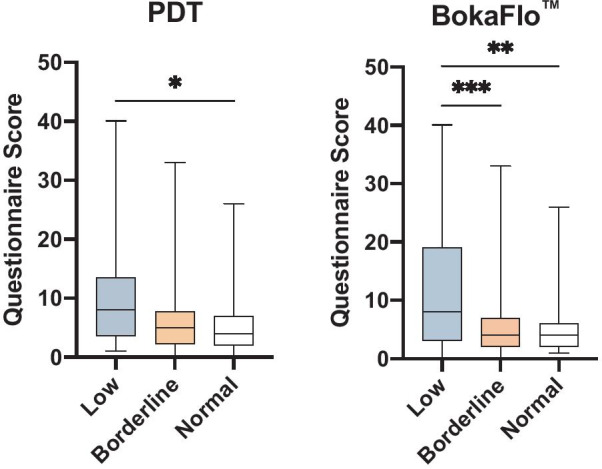
Increased score on oral health questionnaire in participants with low saliva flow as measured by PDT and BokaFlo™. Comparison of the summed questionnaire score for all 15 questions grouped by flow categorization based on both PDT and BokaFlo™. For PDT, a significant difference in mean questionnaire score for low flow and mean questionnaire score for normal flow was present (*p < 0.02). According to BokaFlo™ results, a significant difference in mean was present between both low flow versus borderline questionnaire scores (***p < 0.001) and for low flow versus normal questionnaire scores (**p < 0.002)

Correlative analysis comparing oral health questionnaire results to the BokaFlo™ and PDT saliva flow measures indicated a significant negative correlation between the total oral health questionnaire score and the likelihood of participant exhibiting low salivary flow (r = − 0.31, *p* < 0.006, Fig. 5). Additionally, a significant negative correlation was identified between specific questions on the oral health questionnaire and saliva flow as measured by PDT or BokaFlo™ test (Table 2), including the increased frequency of subjective dry mouth reported by participants (PDT, r = − 0.33, *p* < 0.005; BokaFlo™, r = − 0.35, *p* < 0.005). A significant increase in the oral health questionnaire score was identified in participants with low salivary flow as measured by PDT and BokaFlo™ (Fig. 6).

**Table 2:** Oral Health Questionnaire identified questions that significantly correlated with unstimulated saliva flow

	PDT (Pearson R)	PDT (*p* value)	BokaFlo™ (Pearson R)	BokaFlo™ (*p* value)
My Mouth Feels Dry	− 0.33	0.0032**	− 0.35	0.0017**
I Have Difficulty Swallowing Certain Foods	− 0.22	0.048*	− 0.20	0.086
I Sip Liquids to Aid in Swallowing Food	− 0.21	0.063	− 0.27	0.017*
I Have Difficulty Talking Due to Dry Mouth	− 0.25	0.028*	− 0.23	0.046*
I Drink More During the Day Due to Dry Mouth	− 0.28	0.012*	− 0.34	0.0023**
My Lips Feel Dry	− 0.19	0.086	− 0.28	0.013*
Frequency of Bleeding Gums in the Last 12 Months	0.03	0.81	− 0.23	0.039*
Frequency of Tooth Sensitivity in the Last 12 Months	− 0.13	0.26	− 0.29	0.0096**

*p < 0.05; **p < 0.01

## Discussion

This study demonstrated the ability of the BokaFlo™ instrument to measure hyposalivation in alignment with the PDT methodology, with a sensitivity of 0.76 and a specificity of 0.84. The sensitivity and specificity data and linear regression analysis suggest that the BokaFlo™ device tends to slightly underestimate saliva flow as compared to the PDT. This led to 4 of 35 subjects (11%) that were measured in the healthy range according to PDT to be placed into the hyposalivation category using the BokaFlo™ methodology. Conversely, 2 of 17 subjects (12%) that were measured in the hyposalivation category with the PDT were measured as healthy by BokaFlo™. The differences in measurement of saliva flow by the BokaFlo™ instrument relative to the PDT could be attributed to ability of the sponge applicator to absorb thick or viscous saliva or conformity to differences in sublingual space.

Select questions from the oral health questionnaire significantly correlated with a lower PDT or BokaFlo™ measurement. According to Table 2, the score of questions “My Mouth Feels Dry”, “I Have Difficulty Talking Due to Dry Mouth” and “I Drink More During the Day Due to Dry Mouth” had a significant correlation to low flow in both testing methods. Future oral health questionnaires could be more effective by integrating these identified questions.

Additional methods for measuring salivary flow exist, yet have limitations. When comparing the BokaFlo™ instrument to similar devices, including the Mucus® fourth-generation oral moisture-checking device (OMCD) (Life Co. Ltd.), the BokaFlo™ exhibited a higher sensitivity (0.76) and a similar specificity (0.83) when compared to the PDT. The OMCD had a sensitivity and specificity of 0.65 and 0.83 respectively [20]. The Salimetrics oral swab is another commercial device that can be used for saliva collection and measurement of flow [21]. This method, however, relies on a swab being left in the mouth for 2 min which may stimulate additional saliva flow during collection or possibly irritate the mucosa upon removal of the swab [22]. Suction based methods rely on negative pressure to remove saliva from the mouth as it is secreted. It is possible that such negative pressure may exude additional saliva from glands, or that the presence of a suction device inside the mouth for an extended time may stimulate additional saliva release [14].

In summary, the BokaFlo™ test was much easier to administer than the PDT as it is less time-intensive and increased patient compliance. The ease, comfort, and speed of application could likely lead to higher patient satisfaction rates within the dental clinic [23]. Furthermore, clinicians may be more apt to use the device each visit due to its expediency and comfort for their patients over the PDT methodology.

## Conclusion

This study demonstrated the BokaFlo™ was able to accurately identify individuals with a low salivary flow, evident by the sensitivity and specificity when compared to the gold standard PDT. The total oral health questionnaire score yielded a negative correlation between oral health score and the participants’ saliva flow rate. A select subset of questions on the oral health questionnaire were more effective at indicating whether the participant may have low saliva flow to trigger further evaluation in the dental clinic.

## Supplementary Information

**Additional file 1**. Oral health questionnaire.

## Data Availability

The data generated and analyzed during the current study are available from the corresponding author on reasonable request.

## Abbreviations

PDT: Passive drool test
OMCD: Mucus® fourth-generation oral moisture-checking device
