# Supplementary material for: The use of BokaFlo™ instrument to measure salivary flow
Source: BMC Oral Health. 2021 Apr 12;21:191. doi: 10.1186/s12903-021-01477-4 (PMC8042968; doi:10.1186/s12903-021-01477-4)
Supplement: Supplementary file 1 — Additional file 1. Oral health questionnaire. [file 12903_2021_1477_MOESM1_ESM.pdf]

Supplementary Material

Participant Survey

Pre-Testing Survey

Gender: **Male** **Female**

Age:__________

Did you eat, drink, chew gum, brush teeth or suck on candies in the past 15 minutes? **Yes / No**

Do you currently take any prescription medication? **Yes / No**

How many hours since you last ate food? **Less than 1 hour** **1** **2** **3** **4+ hours**

Do you think you have dry mouth? **Yes / No**

Have you ever been diagnosed by a dentist or doctor with Dry Mouth? **Yes / No**

Do you think you have healthy saliva flow – **Yes / No / I don't know**

Please note the frequency of the following statements:

	Not at all	A little	Quite a bit	Very Much
**My mouth feels dry**	☐	☐	☐	☐
**My mouth feels dry when eating**	☐	☐	☐	☐
**I have difficulty in eating dry food**	☐	☐	☐	☐
**I have difficulties swallowing certain foods**	☐	☐	☐	☐
**I sip liquids to aid in swallowing food**	☐	☐	☐	☐
**I have difficultly talking due to dry mouth**	☐	☐	☐	☐
**I drink more during the day due to dry mouth**	☐	☐	☐	☐
**I have difficulty sleeping due to dry mouth**	☐	☐	☐	☐
**My lips feel dry**	☐	☐	☐	☐

Using the same scale, have had any of the following issues **during the last 12 months:**

	Not at all	A little	Quite a bit	Very Much
**Bleeding gums**	☐	☐	☐	☐
**Cavities**	☐	☐	☐	☐
**Bad breath**	☐	☐	☐	☐
**Frequent cough**	☐	☐	☐	☐
**Frequent colds**	☐	☐	☐	☐
**Tooth sensitivity**	☐	☐	☐	☐
